# Optical Limiting and Femtosecond Pump-Probe Transient Absorbance Properties of a 3,5-distyrylBODIPY Dye

**DOI:** 10.3389/fchem.2019.00740

**Published:** 2019-10-31

**Authors:** Bokolombe P. Ngoy, Aviwe K. May, John Mack, Tebello Nyokong

**Affiliations:** ^1^Department of Chemistry, Institute for Nanotechnology Innovation, Rhodes University, Makhanda, South Africa; ^2^Département de Chimie, Université de Kinshasa, Kinshasa, Democratic Republic of the Congo

**Keywords:** BODIPY dyes, optical limiting, knoevenagel condensation, Z-scan, transient absorbance spectroscopy

## Abstract

The optical limiting (OL) properties of a 3,5-di-*p*-benzyloxystyrylBODIPY dye with an *p*-acetamidophenyl moiety at the *meso*-position have been investigated by using the open-aperture Z-scan technique at 532 nm with 10 ns laser pulses. There is a ca. 140 nm red shift of the main spectral band to 644 nm relative to the corresponding BODIPY core dye, due to the incorporation of *p*-benzyloxystyryl groups at the 3,5-positions. As a result, there is relatively weak absorbance across most of the visible region under ambient light conditions. Analysis of the observed reverse saturable absorbance (RSA) profiles demonstrates that the dye is potentially suitable for use in optical limiting applications as has been reported previously for other 3,5-distyrylBODIPY dyes. Time-resolved transient absorption spectroscopy and kinetic studies with femtosecond and nanosecond scale laser pulses provide the first direct spectral evidence that excited state absorption (ESA) from the S_1_ state is responsible for the observed OL properties.

## Introduction

Non-linear optics (NLO) is a field that focuses on changes in the optical properties of materials upon interacting with intense incident laser pulses. One aspect of NLO that has gained considerable interest over the past few decades is the development of optical limiting (OL) materials that are capable of significantly attenuating the transmittance of light at high incident intensities while remaining optically transparent under ambient light conditions. This normally involves multiphoton processes such as two-photon absorption (2PA), a third-order non-linear process of materials. Since nanosecond laser pulses are used in this study, excited state absorption (ESA) from either the S_1_ or T_1_ states can also result in an OL response. If the ESA is more intense than absorption from the ground state at the wavelength of the incident pulsed laser beam, a solution of the molecular dye absorbs more strongly once the S_1_ and/or T_1_ states are populated after it has interacted with an incident laser pulse (Saleh and Teich, 1991; Tutt and Boggess, 1993; de la Torre et al., 2004). Optical limiting materials can be formed using molecular dyes and other materials that can be used to protect light-sensitive objects such as the human eye and optical sensors by attenuating intense incident laser beams (Dini and Hanack, 2003; Chen et al., 2005). The molecular dyes that have been used as optical limiters typically have a delocalized π-conjugation system which is highly polarizable when interacting with intense laser light (Kandasamy et al., 1997; Ogawa et al., 2002; de la Torre et al., 2004). Phthalocyanines and porphyrins have been the main compounds of interest in this context (Calvete et al., 2004; Chen et al., 2005; Senge et al., 2007), while until recently there had been relatively little research reported on boron-dipyrromethene (BODIPY) dyes in this context (Zhu et al., 2008; Zheng et al., 2009, 2018; Frenette et al., 2014; Kulyk et al., 2016, 2017; Thakare, 2017). Over the past 2 years, we have demonstrated that 3,5-distyrylBODIPY dyes exhibit strong OL responses on the nanosecond timescale (Ndebele et al., 2019).

BODIPY dyes are structural analogs of the porphyrins and consist of two pyrrole units linked by a methene bridge and a BF_2_ moiety. These dyes are structurally versatile due to the stability of the highly robust BODIPY core, allowing facile structural modification for the enhancement of selected properties depending on the potential application of the dye (Loudet and Burgess, 2007; Ulrich et al., 2008; Lu et al., 2014). The second harmonic for Nd:YAG laser beams lies at 532 nm and is particularly important with respect to OL applications given challenges in fields such as aviation safety due to the ever-growing irresponsible use of laser pointers (Harris et al., 2017). The optical properties of the BODIPY dyes must be modified to be useful in OL applications at 532 nm, since this lies close to the main BODIPY spectral band of BODIPY core dyes. One of the most widely used methods to achieve a red shift of the main BODIPY spectral band toward the NIR region is the introduction of styryl groups at the 3,5-positions (Loudet and Burgess, 2007; Ulrich et al., 2008; Lu et al., 2014). Dyes modified in this fashion have an extended degree of π-conjugation and this results in a shift of the main absorption and fluorescence bands to longer wavelengths, hence achieving two of the necessary requirements for NLO studies at 532 nm: a π-conjugation system that results in high polarizability and a main absorption band that lies well to the red of the second harmonic wavelength for Nd:YAG lasers at 532 nm with minimal absorption across most of the visible region.

**Figure 1:**
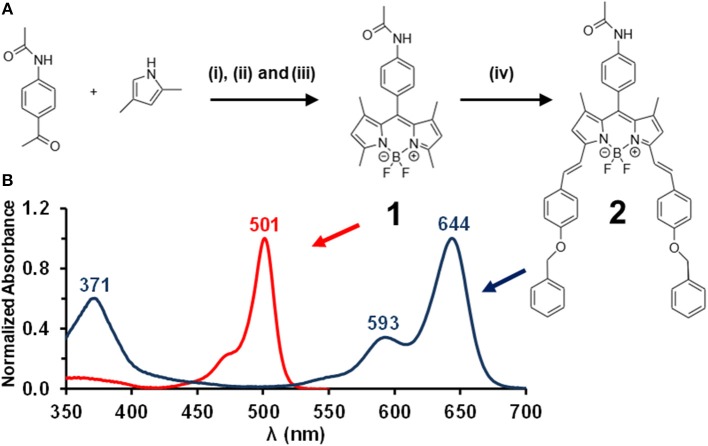
The synthesis of BODIPY dyes **1** and **2 (A)**. Reagents and conditions: (i) TFA at r.t. in dry CH_2_Cl_2_, (ii) *p*-chloranil at 0°C in dry CH_2_Cl_2_, (iii) BF_3_·OEt_2_ and TEA at 0°C in dry CH_2_Cl_2_, and (iv) p-benzyloxybenzaldehyde, piperidine, acetic acid in benzene at reflux with a fitted Dean-Stark trap. Normalized absorption spectra for **1** (red) and **2** (blue) in CH_2_Cl_2_ **(B)**.

The main aim of this study is to further investigate the mechanism that is responsible for the observed NLO properties of 3,5-divinyl- and 3-5-distyrylBODIPY dyes at 532 nm on the nanosecond timescale (Harris et al., 2017; Kubheka et al., 2017, 2018; May et al., 2018; Ngoy et al., 2018). Since these dyes possess an extended π-system, an enhanced NLO response has been reported even in the absence of heavy atoms that promote intersystem crossing to the triplet manifold, in contrast with what has been found with phthalocyanines where the incorporation of heavy atoms has been found to significantly enhance the optical limiting properties in the context of nanosecond laser pulses (Dini and Hanack, 2003). For this reason, we report the first study of the femtosecond pump-probe transient absorbance properties of a novel 3,5-distyrylBODIPY dye (Figure 1A), so that the ESA properties of the singlet manifold can be explored.

## Methods

### Materials

4-Acetamidobenzaldehyde, 4-bromobenzaldehyde, N-bromosuccinimide (NBS), boron trifluoride diethyl etherate (BF_3_·OEt_2_), 2,4-dimethylpyrrole, glacial acetic acid, piperidine, Rhodamine 6G, anhydrous sodium sulfate (Na_2_SO_4_), tetrachloro-1,4-benzoquinone (*p*-chloranil), triethylamine (TEA), trifluoroacetic acid (TFA), and zinc phthalocyanine were purchased from Sigma-Aldrich. Spectroscopic grade solvents were used for the photophysical and the open aperture Z-scan studies.

### Instrumentation

^1^H NMR data were measured on a Bruker AMX 600 NMR instrument. Mass spectral data were recorded with a Bruker AutoFLEX III Smart beam TOF/TOF Mass spectrometer. The spectra were acquired using α-cyano-4-hydroxycinnamic acid as the MALDI matrix, and a 355 nm Nd:YAG laser as the ionizing source. UV-visible absorption spectra were recorded on a Shimadzu UV-2550 spectrophotometer, and infrared spectra were measured with a Perkin Elmer Spectrum 100 FT-IR spectrometer. Fluorescence emission spectra were recorded with a Varian Eclipse instrument, while fluorescence lifetime (τ_F_) values were calculated by using a Picoquant FluoTime 200 time-correlated single-photon counting instrument. Fluorescence quantum yield (Φ_F_) values were calculated by using the comparative method (Ogunsipe et al., 2004). Standard and sample solutions with identical optical densities were excited at the same wavelength in DMSO, with zinc phthalocyanine used as the standard. Triplicate measurements were made to ensure accuracy.

A frequency-doubled Quanta-Ray Nd:YAG laser was used in a near Gaussian transverse mode to carry out open aperture Z-scan measurements on BODIPY dyes (Ndebele et al., 2019) using an instrumental setup that has been described previously (Neethling, 2005). The 532 nm beam was spatially filtered with Glan Thompson GTH10M polarizers to remove higher-order modes and was tightly focused with a plano-convex Thorlabs LA1433 lens with a 15 cm focal length. To enable the Z-scan measurement the sample was translated along the z-axis direction parallel to the incident laser beam with a Newport M-ILS250CCL translation stage in 0.5 mm steps over a total 80 mm path centered on the focal point of the lens. Coherent J5-09 energy detectors (energy ranges of 0.1 μJ−0.1 mJ) and a Coherent EPM2000 energy meter were used to determine normalized transmittance values. A Thorlabs BSW07 beam splitter enables the measurement of both the incident intensity and that transmitted through the sample. Normalized transmittance values determined for 32 consecutive laser pulses were averaged to obtain a value at each z value during the Z-scan measurements. A 2 mm optical glass cuvette was used to obtain the Z-scan data for BODIPY **2** in CH_2_Cl_2_ solution.

Femtosecond transient absorption measurements were made with a Clark MXR CPA2001 titanium-sapphire (Ti/Sa) laser system with a pulse energy of 0.9 mJ, and a full width at half-maximum (FWHM) of 150 fs, and an operating frequency of 426 Hz as has been reported previously (Klíčová et al., 2012) with slight modifications. The pump-supercontinuum probe technique was implemented. Since BODIPY **2** does not absorb in the near infrared region, the output of the Ti/Sa laser at 775 nm was directly frequency-doubled by a β-barium borate (BBO) crystal to 387.5 nm to generate the pump beam. The energy of the laser pulses were 1 μJ energy in this context, while the pulse width was kept below 150 fs. The probe beam was generated by focusing a 2 mm CaF_2_ plate in front of the 775 nm beam to produce a 270–690 nm supercontinuum span. The CH_2_Cl_2_ solution of BODIPY **2** was passed through an optical flow cell with a thickness of 0.4 mm, and the probe and pump beams were focused onto a 0.2 mm spot. The pump beam and a reference signal that was also passed through the solution in the absence of the probe beam were dispersed spectrally and recorded with two 512 pixel photodiode arrays. The ratio of the intensities of the two beams was used to generate the transient absorption spectra. The pump-probe cross-correlation was determined to be <100 fs across the whole spectrum. Measurements on time scales below 50 ps were corrected for chirp in the manner described previously (Klíčová et al., 2012). Data were averaged over three pump-probe scans with 400 shots per temporal point to lower the signal-to-noise ratio.

Nanosecond laser flash photolysis kinetics were studied with an Ekspla NT 342B-20-AW laser that contains an Nd:YAG (355 nm, 78 mJ/7 ns, 20 Hz) pumping a 420–2,300 nm range optical parametric oscillator (8 mJ/7 ns, 20 Hz) to provide the pump beam for an Edinburgh Instruments LP980 transient absorption spectrometer. A 150 W Xe arc lamp provided the probe beam and was used in its continuous mode. The LP980 instrument is fitted with a Quantum Composers 9512+ pulse generator and a Tektronix TDS 3012C oscilloscope to collect data under the control of Edinburgh Instruments' L900 software. The spectrometer contains 500 nm blazed gratings for both excitation and emission and is fitted with a PMT-LP (Hamamatsu R928P) and an ICCD camera (Andor DH320T-25F03). The CH_2_Cl_2_ solution of **2** was excited with the pump beam at the band maximum of the main BODIPY spectral band at 644 nm. Samples were deoxygenated with inert nitrogen gas.

### Synthesis

An adapted version of the conventional 1-pot 3-step reaction procedure for BODIPY synthesis (Yogo et al., 2005) was used to prepare BODIPY **1** (Figure 1A). BODIPY **1** was then used as a precursor to form 3,5-distyryl BODIPY **2** (Figure 1A) through a Knoevenagel condensation reaction (Rurack et al., 2001) by introducing the *p*-benzyloxystyryl groups by reacting **1** with *p*-benzyloxybenzaldehyde. TLC plates were used to verify the completion of the reaction through the absence of unreacted BODIPY core **1**, and to confirm after purification of **2** by column chromatography that other side products are not present. No additional UV-visible absorption bands were observed due to unwanted side products. The MALDI-TOF MS data for **2** and ^1^H NMR spectra obtained for **1** and **2** were found to be fully consistent with the anticipated structures.

#### 4,4′-Difluoro-8-(4-acetamidophenyl)-1,3,5,7-tetramethyl-4-bora-3a,4a-diaza-s-indacene (1)

A solution of 2,4-dimethylpyrrole (2 eq) and 4-acetamidobenzaldehyde (1 eq) was prepared in dry CH_2_Cl_2_ (50 mL) under inert Ar gas. Two to three drops of TFA were added, followed by stirring at room temperature. TLC was used to confirm the complete consumption of the aldehyde. A *p*-chloranil solution (1.2 eq) in dry CH_2_Cl_2_ (10 mL) was then added at 0°C, and the reaction mixture was stirred for 30 min at room temperature under Ar gas. A deep purple color was observed, and TLC confirmed the synthesis of the dipyrromethene. TEA (7 eq) was added dropwise at 0°C and BF_3_·OEt_2_ (11 eq) was then added in a similar manner, followed by stirring for 12 h at room temperature. The reaction mixture was filtered, washed with water (100 mL), and dried over anhydrous Na_2_SO_4_. Silica gel column chromatography with ethyl acetate:petroleum ether (1:4) as the eluent provided the pure target compound in 33% yield. ^1^H NMR (600 MHz, CDCl_3_) δ_H_, ppm 7.70 (d, J = 7.9 Hz, 2H), 7.47 (s, 1H), 7.24 (d, J = 7.9 Hz, 2H), 6.00 (s, 2H), 2.57 (s, 6H), 2.25 (s, 3H), 1.44 (s, 6H). FT-IR: ν, cm^−1^ 3,368 (N-H amide stretch), 2,920 (C-H stretch), 2,852 (C-H stretch), 1,529 (N-H amide bend), 1,459, 1,399 (C-N stretch), 1,186 (C-N stretch), 967 (= C-H bend), 470 (C-H).

#### 4,4′-Difluoro-8-(4-acetamidophenyl)-1,7-dimethyl-3,5-di-(4-benzyloxy)styryl-4-bora-3a,4a-diaza-s-indacene (2)

Piperidine (1 mL) and glacial acetic acid (1 mL) were added to a solution of 4-benzyloxybenzaldehyde (2 eq) and **1** (1 eq) in benzene (50 mL). The reaction mixture was heated at reflux for several hours, and water was removed with a Dean-Stark trap. The solvent was removed under vacuum on a rotary evaporator and the crude product was diluted in CH_2_Cl_2_, washed with water and then dried over anhydrous Na_2_SO_4_. The solvent was removed under vacuum and pure target compound was obtained in 32% yield by silica gel column chromatography with ethyl acetate:petroleum ether (1:4) as the eluent. ^1^H NMR (600 MHz, CDCl_3_) δ_H_, ppm 7.70 (d, J = 7.9 Hz, 2H), 7.64–7.58 (m, 6H), 7.51–7.46 (m, 5H), 7.46–7.42 (m, 4H), 7.39–7.35 (m, 2H), 7.27–7.21 (m, 4H), 7.02 (d, J = 8.2 Hz, 4H), 6.62 (s, 2H), 5.13 (s, 4H), 2.24 (s, 3H), 1.48 (s, 6H). FT-IR: ν, cm^−1^ 3,377 (N-H amide stretch), 2,916 (C-H stretch), 2,853 (C-H stretch), 1,471 (N-H amide bend), 1,368 (C-N stretch), 1,103 (C-N stretch), 1,154 (C-O-C), 979 (= C-H bend). MS (MALDI-TOF): m/z 769.30 (calc. for [M]^+^ 769.86).

## Results and Discussion

A typical 3,5-distyrylBODIPY dye was synthesized so that its photophysical and optical limiting properties could be investigated. A *p*-acetamidophenyl group was introduced at the *meso*-position to form a novel BODIPY core dye **1** (Figure 1A) and benzyloxystyryl moieties were added at the 3,5-positions to form a novel 3,5-distyrylBODIPY dye **2**. We have previously reported the optical limiting properties of 3,5-di-*p*-benzyloxystyrylBODIPY dyes with a range of different *meso*-substituents (Ngoy et al., 2018; Ndebele et al., 2019). The results were found to be broadly similar and to suggest that these dyes are potentially suitable for use as OL materials. In this study, transient absorbance spectroscopy and kinetic studies with femtosecond and nanosecond laser pulses are used to investigate the role of ESA from the S_1_ state in the context of 3,5-distyrylBODIPY dyes. Since there are no heavy atoms incorporated, these dyes are not expected to undergo significant intersystem crossing to the triplet manifold.

### Optical Spectroscopy and Photophysical Properties

The UV-visible absorption spectra of **1** and **2** (Figure 1B) are typical of what is normally observed for a 1,3,5,7-tetramethylBODIPY core dye and its 3,5-distyryl derivative (Lu et al., 2014). Since the BODIPY chromophore lacks a macrocycle, the visible region is dominated by a single intense spectral band and does not give rise to the Q and B bands that dominate the optical spectra of porphyrins and phthalocyanines (Lu et al., 2014). The main absorption band of BODIPY core dye **1** lies at 501 nm in CH_2_Cl_2_, while that of **2** lies at 644 nm with a log ε value of 5.56 in CH_2_Cl_2_. The main emission bands of **1** and **2** lie at 514 and 660 nm, respectively, and were found to have Φ_F_ values of 0.50 and 0.40 and τ_F_ values of 3.3 and 4.6 ns (Table 1). **2** is hence moderately fluorescent, in a similar manner to what has previously been observed for a wide range of BODIPY dyes that have extended π-systems with co-planar styryl or vinylene substituents (Ndebele et al., 2019). Theoretical calculations have demonstrated that the large red shift of the main BODIPY spectral band of 3,5-distyrylBODIPY dyes upon styrylation results primarily from a relative destabilization of the HOMO (Lu et al., 2014; Ndebele et al., 2019), which has larger MO coefficients at the 3,5-positions at the LUMO. This results in a significant narrowing of the HOMO–LUMO gap. Since a relatively large energy gap is predicted between the S_1_ and S_2_ excited states of 3,5-distyrylBODIPY dyes (Ndebele et al., 2019), these dyes exhibit only relatively weak absorbance under ambient light conditions across most of the visible region (Figure 1B). These properties make 3,5-distyrlBODIPY dyes suitable for use in OL applications in the context of visible region laser pulses.

**Table 1:** Photophysical data for BODIPYs **1** and **2** in CH_2_Cl_2_, and optical limiting parameters for **2** in CH_2_Cl_2_.

**Photophysical properties**						
	**λ_Abs_** **(nm)**	**λ_ex_** **(nm)**	**λ_em_** **(nm)**	**Φ_F_**	**τ_F_** **(ns)**	
**1**	501	500	514	0.50	3.3	
**2**	644	644	660	0.40	4.6	
**Optical limiting properties**					
	**Energy (μJ)**	**α** **(cm**^**−1**^**)**	**β_eff_** **(cm.GW**^**−1**^**)**	**Im[χ^(3)^] (esu)**	**γ** **(esu)**	**I**_**lim**_ **(J.cm**^**−2**^**)**
**2**	30	0.78	145.5	3.17 ×10^−10^	3.25 ×10^−29^	0.80

### Optical Limiting Properties

**Figure 2:**
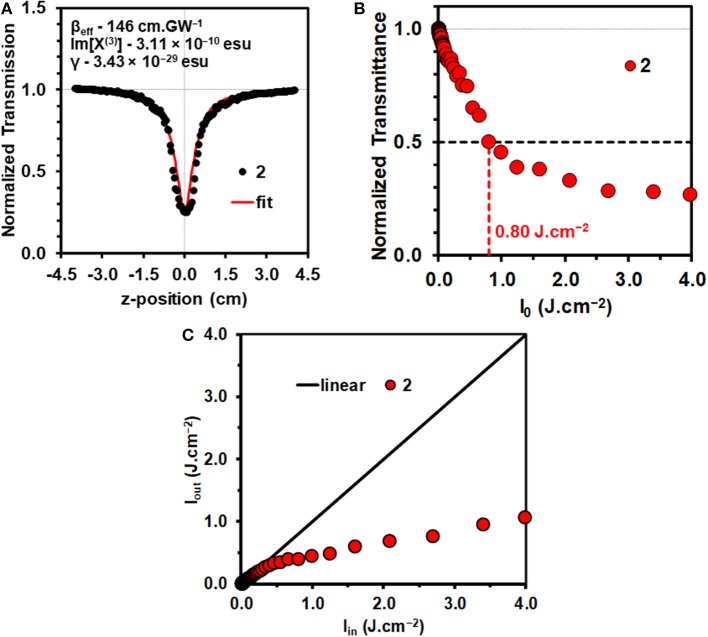
Open-aperture Z-scan for a 4.9 × 10^−6^ M solution of **2** in CH_2_Cl_2_ at an input intensity of 32 μJ with the calculated NLO parameters **(A)**. Normalized transmittance vs. input fluence (I_in_) curve for **2** in CH_2_Cl_2_ **(B)**. The calculation of the I_lim_ value is shown with horizontal and vertical lines. Output fluence (I_out_) vs. input fluence (I_in_) curves for **2 (C)**. Details of the optical limiting parameters are provided in Table 1.

The mechanisms that can result in an optical limiting effect are non-linear absorption (NLA), non-linear refraction (NLR), and non-linear scattering (NLS). Since a solution of a molecular dye is involved in the context of this study, NLA is the dominant mechanism. Z-scan measurements were carried out for **2** in a 4.9 × 10^−6^ M CH_2_Cl_2_ solution (Figure 2A) with 10 ns laser pulses. The concentration used lies within the linear range according to the Beer-Lambert law. This prevents aggregation that can result in significant NLS.

Since nanosecond laser pulses were used in this study, the concave downward dipping reverse saturable absorption (RSA) response (Figure 2A) that is observed for **2** in the open aperture Z-scan data is unlikely to arise exclusively from multiphoton processes such as 2PA. ESA from the S_1_ or T_1_ state can result in an RSA response when the ESA is more intense than the ground state absorbance, due to the effect of depopulating the ground state (Saleh and Teich, 1991; Tutt and Boggess, 1993; de la Torre et al., 2004), so only a β_eff_ value can be quantified for the non-linear absorption coefficient rather than the intrinsic β value that is associated with 2PA. There have been a limited number of studies of the intrinsic β values of BODIPY dyes in femtosecond pump-probe studies (Wang et al., 2010; Kim et al., 2015; Zheng et al., 2018).

The normalized transmittance values at each z-axis position [T(z)] were analyzed as the sample is translated through the focal point of the lens used in the Z-scan measurements by using the approach developed by Sheik-Bahae et al. (1989, 1990) and Sheik-Bahae and Van Stryland (1998) (Equations 1–4):

(1) $$\begin{array}{l} \text{T}(\text{z})=\frac{1}{\sqrt{\;\pi \;}{\text{q}}_{0}(\text{z})}\int_{\text{-}\infty }^{\infty }\text{ln}\left[1+{\text{q}}_{0}(\text{z}){\text{e}}^{-{\;\tau \;}^{2}}\;\right]\;d\tau \end{array}$$

where the size of the non-linear response is described by q_0_(z). For a circular beam, the q_0_(z) value is described by Equation 2:

(2) $$\begin{array}{l} {\text{q}}_{0}(\text{z})=\frac{2\beta_{\text{eff}}{\text{P}}_{0}{\text{l}}_{\text{eff}}}{\;\pi \;w(\text{z}{)}^{2}} \end{array}$$

where P_0_ is the peak power of the laser pulse, β_eff_ is the effective non-linear absorption coefficient, and l_eff_ is the effective pathlength, given by Equation 3:

(3) $$\begin{array}{l} {\text{l}}_{\text{eff}}=\frac{1-e^{\text{(}-\;\alpha \text{L})}}{\;\alpha \;} \end{array}$$

where L is the pathlength. The linear absorption coefficient, α, is described by Equation 4:

(4) $$\begin{array}{l} \;\alpha \;=\frac{2.303\cdot \text{OD}}{\text{L}} \end{array}$$

where OD is the optical density of the solution at 532 nm. The beam width [w(z)], in Equation 2 can be described as a function of sample position using Equation 5:

(5) $$\begin{array}{l} \text{w}(\text{z})={\text{w}}_{0}\sqrt{1+{\left(\frac{\text{z}}{{\text{z}}_{0}}\right)}^{2}} \end{array}$$

where z is the translation distance of the sample from the laser focal point, and z_0_ is the Rayleigh length that is defined as π${\text{w}}_{0}^{2}$/λ where λ is the wavelength of the laser, w_0_ is the beam waist value at the focal point (z = 0) of the laser beam which describes the distance where there is 1/e^2^ intensity relative to the on axis value.

Although the effective non-linear absorption coefficient, β_eff_, can be described using Equations 1–5, the q_0_(z) value is usually derived from the normalized transmittance data by applying an analytical version of Equation 1 (Ndebele et al., 2019):

(6) $$\begin{array}{l} \text{T}(\text{z})=0.363{\text{e}}^{\left(\frac{-{\text{q}}_{0}(\text{z})}{5.60}\right)}+0.286{\text{e}}^{\left(\frac{-{\text{q}}_{0}(\text{z})}{1.21}\right)}+0.213{\text{e}}^{\left(\frac{-{\text{q}}_{0}(\text{z})}{24.62}\right)}\\ \;+0.096{\text{e}}^{\left(\frac{-{\text{q}}_{0}(\text{z})}{115.95}\right)}+0.038{\text{e}}^{\left(\frac{-{\text{q}}_{0}(\text{z})}{965.08}\right)} \end{array}$$

When Equation 2 is substituted into Equation 5, the q_0_(z) value can be described as:

(7) $$\begin{array}{l} {\text{q}}_{0}(\text{z})=\frac{{\text{Q}}_{0}}{1+\frac{{\text{z}}^{2}}{{\text{z}}_{0}^{2}}} \end{array}$$

where Q_0_ is given as:

(8) $$\begin{array}{l} {\text{Q}}_{0}=\frac{2\beta_{\text{eff}}{\text{P}}_{0}{\text{l}}_{\text{eff}}}{\;\pi \;w{\;}_{0}^{2}} \end{array}$$

The FWHM and peak maximum values for the Gaussian curve that is defined by Equation 1 are proportional to the z_0_ and Q_0_ values as defined by Equations 7, 8. Equation 9 is used to determine the β_eff_ value, which is dependent on the population of molecules in the excited state. The magnitude of the β_eff_ value provides an indication of how suitable materials are for use in optical limiting applications:

(9) $$\begin{array}{l} \beta_{\text{eff}}=\frac{\;\lambda \;{\text{z}}_{0}{\text{Q}}_{0}}{2{\text{P}}_{0}{\text{l}}_{\text{eff}}} \end{array}$$

The imaginary component of the third-order non-linear susceptibility, Im[χ^(3)^], provides a measure of how rapidly an OL material responds to perturbations that are initiated by intense incident laser pulses (Sheik-Bahae and Van Stryland, 1998; Dini and Hanack, 2003). The relationship between the Im[χ^(3)^] and β_eff_ values is described by Equation 10:

(10) $$\begin{array}{l} \text{Im}\left[{\;\chi \;}^{\text{(3)}}\right]=\frac{{\;\eta \;}^{2}{\;\varepsilon \;}_{0}\text{c}\lambda \;\beta_{\text{eff}}}{2\pi \;} \end{array}$$

Where ε_0_, η, and c are the permittivity of free space, the linear refractive index and speed of light, respectively.

The interaction between the permanent dipole of molecules with incident laser beams of high intensity can modulate the average orientation of the molecules in a manner that also induces second-order hyperpolarizability, γ. The relationship between the γ and Im[χ^(3)^] values is shown in Equation 11:

(11) $$\begin{array}{l} \;\gamma \;=\frac{\text{Im}\left[{\;\chi \;}^{\text{(3)}}\right]}{{\text{f}}^{4}{\text{C}}_{\text{mol}}{\text{N}}_{\text{A}}} \end{array}$$

where N_A_, f, and C_mol_ are Avogadro's constant, the Lorentz local field factor (f = (η^2^+2)/3), and the molar concentration of the active species, respectively.

The data were analyzed in a similar manner to what has been described previously (Harris et al., 2017; Kubheka et al., 2018; Ngoy et al., 2018, 2019; Ndebele et al., 2019) to determine the β_eff_, Im[χ^(3)^], and γ values. A linear absorption coefficient, α, of 0.78 cm^−1^ was obtained at 532 nm for the 4.9 × 10^−6^ M CH_2_Cl_2_ solution of **2** that was studied (Table 1), so the S_1_ state is likely to be populated primarily by linear absorption rather than through multiphoton processes. The β_eff_ value obtained for **2** (Figure 2A and Table 1) provides a measure of the magnitude of the non-linear absorptivity, and lies within the range previously reported for other organic compounds (Dini and Hanack, 2003; Sutherland et al., 2003). OL materials have positive β_eff_ values since there is a marked decrease in transmittance at the focal point of the Z-scan instrument.

The γ and Im[χ^(3)^] values that were obtained (Table 1) confirm that BODIPY **2** has promising OL properties, since they lie in the 10^−29^–10^−34^ and 10^−9^–10^−15^ esu ranges (Dini and Hanack, 2003; Sutherland et al., 2003), respectively, that have previously been reported to be favorable for use in optical limiting in the context of molecular dyes, such as porphyrins and phthalocyanines (de la Torre et al., 2004; Senge et al., 2007; Dini et al., 2016). A review was recently published that provides the β_eff_, γ and Im[χ^(3)^] values for 17 different π-expanded BODIPY dyes on the nanosecond timescale at 532 nm (Ndebele et al., 2019). No comparison can be made with femtosecond timescale data for BODIPYs, since a multiphoton absorption mechanism is involved in that context in the absence of ESA (Wang et al., 2010; Kim et al., 2015; Zheng et al., 2018). The γ value is the most useful parameter for making direct comparisons between the OL properties, since Equation 11 includes the concentration, while Equations 9 and 10 do not since the β_eff_ and Im[χ^(3)^] values are concentration dependent. The γ value for **2** (3.25 × 10^−29^) is one of the highest we have reported to date for a 3,5-distyryl- or 3,5-divinyleneBODIPY and is comparable in magnitude to the value of 9.6 × 10^−30^ esu that was recently reported for a 3,5-di-*p*-benzyloxystyrylBODIPY with a *p*-hydroxyphenyl group at the *meso*-position (Ngoy et al., 2018). The γ values of most 3,5-distyrylBODIPYs have been reported to lie in the 10^−30^ esu range (Ndebele et al., 2019).

For a material to be considered to be viewed as suitable for OL applications, the transmittance should decrease by over 50% as is the case with dye **2** (Figure 2B). It is important to be able to determine the input energy at which this happens. Irradiance has units of W.cm^−2^, with the maximum value, I_00_, occurring at the focus (z = 0), determined by Equation 12.

(12) $$\begin{array}{l} {\text{I}}_{00}=\frac{\text{E}}{\;\tau \;\pi {\text{w}}_{0}^{2}} \end{array}$$

where w_0_ is the beam waist (cm), τ is the laser pulse length (s), and E is the energy of the laser pulse (J). The maximum irradiance values can be expressed in units of W.cm^−2^, since 1 J.s^−1^ = 1 W. Since the energy of the laser pulses is kept constant during the Z-scan measurements, the overall power of the laser beam is also constant throughout, but the irradiance value will change with the beam width as the sample is moved into and out of focus along the z-axis direction, w(z). The area of the beam is circular and hence equal to πw(z)^2^. Multiplying the circular cross-sectional beam area, πw(z)^2^, by the irradiance gives the laser power, P, at the focal point (z = 0), Equation 13:

(13) $$\begin{array}{l} \text{P}={\text{I}}_{00}\;\pi {\text{w}}_{0}^{2} \end{array}$$

At other z positions, P can be expressed by Equation 14.

(14) $$\begin{array}{l} \text{P}={\text{I}}_{\text{in}}(\text{z})\pi \text{w}(\text{z}{)}^{2} \end{array}$$

Since P is kept constant, Equations 13 and 14 can be combined to provide the input fluence value, Equation 15:

(15) $$\begin{array}{l} {\text{I}}_{\text{in}}(\text{z})={\text{I}}_{00}{\left(\frac{{\text{w}}_{0}}{\text{w}(\text{z})}\right)}^{2} \end{array}$$

Since the transmittance provides the percentage of light that passes through the material, values for output fluence can be determined from the product of T(z) and I_in_(z) at each z position, Equation 16.

(16) $$\begin{array}{l} {\text{I}}_{\text{out}}(\text{z})={\text{I}}_{\text{in}}(\text{z})\text{T}(\text{z}) \end{array}$$

The limiting threshold fluence (I_lim_) can be defined as the input fluence value at which the output fluence (I_out_) is decreased to 50% of the input fluence (I_in_), and this can be readily determined using a plot of normalized transmittance vs. input fluence (Figure 2B). A relatively low I_lim_ value of 0.80 J.cm^−2^ was obtained in CH_2_Cl_2_ solution (Table 1). This provides further evidence that 3,5-distyrylBODIPYs are potentially suitable for use in OL applications. A wide range of different π-expanded BODIPY dyes have provided broadly similar I_lim_ values (Ndebele et al., 2019). Since the limiting threshold can be lowered by increasing the concentration of the solution, I_lim_ values reported in the literature cannot be readily compared. The International Commission on Non-Ionizing Radiation Protection has published a guideline (International Commission on Non-Ionizing Radiation Protection (ICNIRP), 2000) for exposure to a variety of lasers, which has been determined to provide a limit of 0.95 J.cm^−2^ for the 0.25 s exposure time for the normal human blink reflex to prevent significant damage to the human eye (Harris et al., 2017). Plotting I_out_ against I_in_ (Figure 2C) can be used to demonstrate whether the I_out_ values approach a plateau as the I_in_ value increases, which is the type non-linear response that would normally be expected for an OL material that is suitable for practical applications (Dini and Hanack, 2003; Sutherland et al., 2003).

### Pump-Probe Transient Spectroscopy Studies

**Figure 3:**
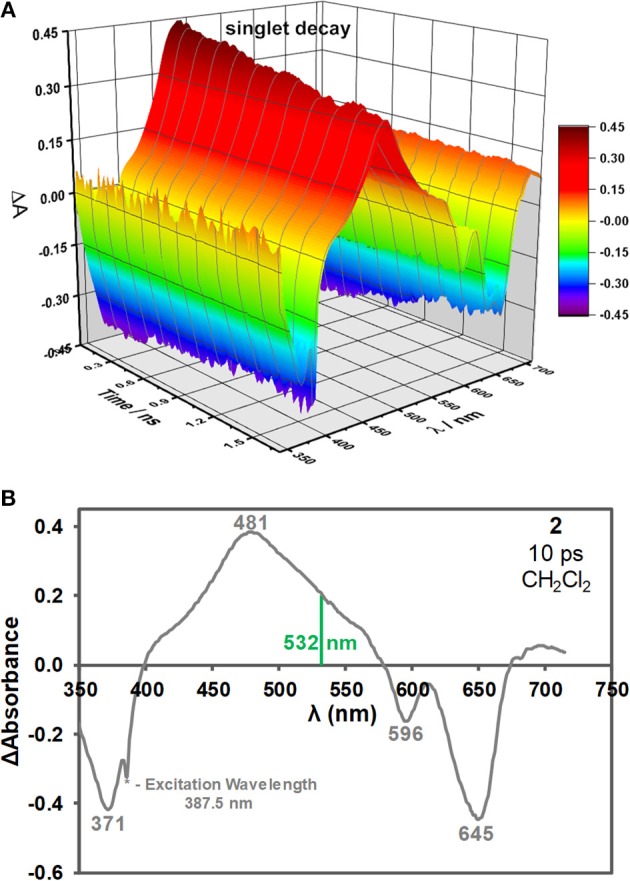
Time-resolved transient absorption spectra of **2** in CH_2_Cl_2_ after a 150 fs laser pulse at an excitation wavelength of 387.5 nm **(A)**. The transient absorption spectrum of **2** at 10 ps after a 150 fs laser pulse at 387.5 nm in CH_2_Cl_2_ **(B)**. The 532 nm wavelength used for the open-aperture Z-scan measurement is highlighted with a green vertical line.

BODIPY **2** is not halogenated and contains no other heavy atom. BODIPY dyes of this type usually have very low triplet state and singlet oxygen quantum yields in the absence of heavy atoms (Yogo et al., 2005; Jiao et al., 2011; Yang et al., 2013; Frenette et al., 2014), so the S_1_ state is believed to be involved with the ESA of non-halogenated 3,5-distyrylBODIPY dyes (Harris et al., 2017; Kubheka et al., 2018; Ngoy et al., 2018, 2019). This was confirmed in this study by carrying out the femtosecond and nanosecond pump-probe spectroscopy and kinetic studies. A time-resolved transient spectroscopic study was carried out for solutions of BODIPY dye **2** (ca. 5.0 × 10^−3^ M) in CH_2_Cl_2_ with a femtosecond laser at 387.5 nm in order to study what happens during Z-scan measurements when the S_1_ state is populated upon electronic excitation (Figure 3A). As would normally be anticipated, intense ground state depletion peaks are observed at 371, 596, and 645 nm, which correspond to the main absorption bands of **2** (Figures 1B, 3B). These signals decay on a nanosecond timescale (Figure 3A) in a manner that is consistent with the observed fluorescence lifetime of 4.6 ns (Table 1), since the decay of the ESA and fluorescence intensities are both dependent upon changes in the population of the S_1_ state after its initial population by an incident laser pulse. An intense broad peak is observed due to ESA across most of the visible region between 400 and 580 nm with a maximum at 481 nm (Figure 3B). When attempts were made to measure transient absorption spectra and decay curves on the microsecond timescale for the T_1_ state of **2** using 7 ns laser pulses to excite at the band maximum at 644 nm (Figure 1B), no decay curve was observed for **2** in degassed CH_2_Cl_2_ solutions. In contrast, data could be readily measured with comparable solutions of halogenated BODIPYs using a similar approach, since the rate of intersystem crossing is greatly enhanced by the heavy atom effect. This demonstrates that ESA associated with the T_1_ state does not play a significant role in the optical limiting properties of **2**.

## Conclusions

The open aperture Z-scan study on a 3,5-di-*p*-benzyloxystyrylBODIPY dye at 532 nm on the nanosecond timescale demonstrates that dyes of this type have favorable OL properties as has been reported previously. Time-resolved transient-absorption spectra were recorded after a 150 fs laser pulse to provide further insights into the mechanism of the observed optical limiting response. A broad and intense band is observed between 400 and 580 nm due to ESA from the S_1_ state, which is consistent with the observed strong RSA response at the 532 nm. In contrast, no triplet decay curve was observed on the microsecond timescale for **2** when 7 ns laser pulses were used instead. This provides the first direct spectroscopic evidence that the OL effects that have consistently been observed at 532 nm for 3,5-divinyl- and 3,5-distyrylBODIPY dyes that contain no heavy halogen atoms at the 2,6-positions are due primarily to ESA from the S_1_ state to higher energy states in the singlet manifold. The key to further enhancing the reverse saturable absorbance response that is observed for nanosecond laser pulses in the visible region is to further enhance this ESA. Since it is usually important that optical limiting materials remain transparent under ambient light conditions, the goal in future will be to identify how the structure of the BODIPY chromophore can be modified in a rational manner to further enhance this ESA, while also shifting the main spectral band of the BODIPY chromophore to the red so that there is minimal absorbance across the entire visible region. The use of hybrid materials in which BODIPY dyes are conjugated with nanomaterials in a similar manner to what has been reported previously for porphyrins and phthalocyanines (Dini et al., 2016) also merits in depth investigation in the years ahead.

## Data Availability Statement

The key data sets that support the conclusions reported in this manuscript will be made available by the corresponding author to any qualified researcher without undue reservation.

## Author Contributions

BN synthesized BODIPYs **1** and **2** and carried out the femtosecond timescale kinetic measurements. AM carried out the Z-scan measurements, the photophysical studies, and the nanosecond timescale kinetic measurements. JM conceptualized the research and was the lead author in preparing the manuscript. TN provided the resources for the project and also assisted with the writing of the manuscript.

### Conflict of Interest

The authors declare that the research was conducted in the absence of any commercial or financial relationships that could be construed as a potential conflict of interest.
